# Supplementary material for: The causal relationship between the human gut microbiota and pyogenic arthritis: a Mendelian randomization study
Source: Front Cell Infect Microbiol. 2024 Nov 26;14:1452480. doi: 10.3389/fcimb.2024.1452480 (PMC11629706; doi:10.3389/fcimb.2024.1452480)
Supplement: Supplementary file 1 [file DataSheet1.zip › mendelian test/ebi-a-GCST90027640.csv_forest.pdf]

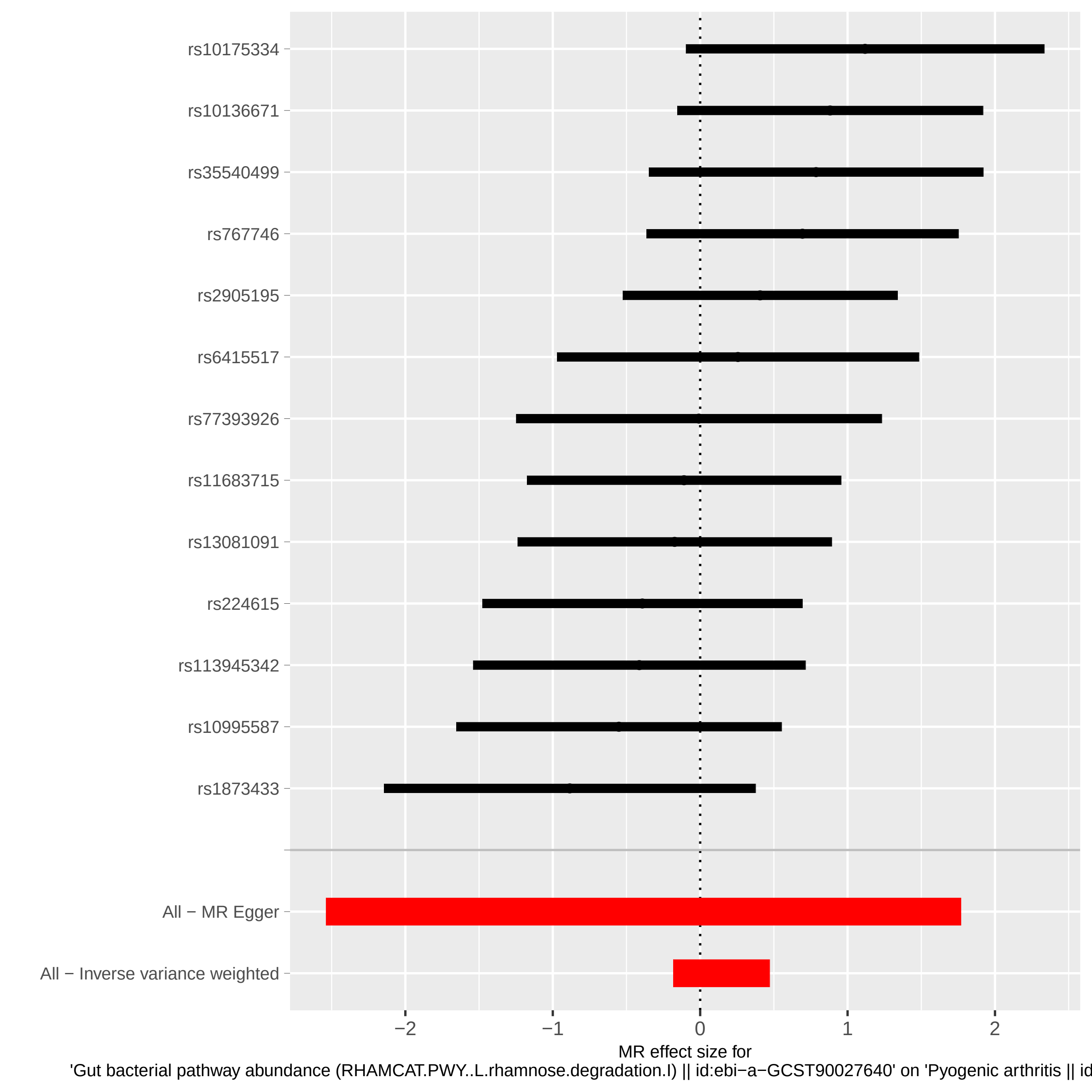

rs10175334
rs10136671
rs35540499
rs767746
rs2905195
rs6415517
rs77393926
rs11683715
rs13081091
rs224615
rs113945342
rs10995587
rs1873433
All − MR Egger
All − Inverse variance weighted
−2
−1
0
1
2
MR effect size for
'Gut bacterial pathway abundance (RHAMCAT.PWY..L.rhamnose.degradation.I) || id:ebi−a−GCST90027640' on 'Pyogenic arthritis || id
